# Developing and Validating a Big-Store Multiple Errands Test

**DOI:** 10.3389/fpsyg.2019.02575

**Published:** 2019-11-15

**Authors:** Kristen Antoniak, Julie Clores, Danielle Jensen, Emily Nalder, Shlomit Rotenberg, Deirdre R. Dawson

**Affiliations:** ^1^Department of Occupational Science & Occupational Therapy, University of Toronto, Toronto, ON, Canada; ^2^Alberta Health Services, Calgary, AB, Canada; ^3^Rehabilitation Sciences Institute, University of Toronto, Toronto, ON, Canada; ^4^Rotman Research Institute, Baycrest Health Sciences, Toronto, ON, Canada

**Keywords:** Multiple Errands Test, executive functioning, cognition, community, ecological validity

## Abstract

The Multiple Errands Test (MET) is an ecologically valid assessment that characterizes how executive dysfunction manifests in everyday activities. Due to the naturalistic nature of this assessment, clinicians and researchers have had to develop site-specific versions resulting in numerous published versions and making it difficult to establish standard psychometric properties. The aim of this study was to develop a standardized, community version of the MET designed to be used in large department stores meeting set criteria that would not require site specific modifications. This paper reports on the development, content validity, feasibility, and inter-rater reliability of a Big-Store MET, and the performance of healthy participants on this test. Items were selected to match previously published versions in relation to quantity and complexity. Content validity was established by having experts (*n* = 4) on the MET review the proposed Big-Store version and evaluate the task consistency with previously published versions. To assess feasibility of administration, and inter-rater reliability, a convenience sample of 14 community dwelling adults, self-reporting as healthy, were assessed by two trained raters. We found the Big-Store MET to be feasible to deliver (completed within 30 min, scores show variability, acceptable to participants in community environment) and inter-rater reliability to be very high (ICCs = 0.92–0.99) with the exception of frequency of strategy use. This study introduces the Big-Store MET to the literature, establishes its preliminary validity and reliability thus laying the foundation for a standardized, community-based version of the MET.

## Introduction

The Multiple Errands Test (MET) was originally developed by Shallice and Burgess (1991) in response to their (and others) observations that some patients with frontal lobe lesions often perform relatively well on standardized neuropsychological tests while having significant difficulties in everyday life. Shallice and Burgess (1991) noted that the structure and administration of standardized neuropsychological tests rarely provide opportunities for people to plan and organize their behavior for more than a few minutes nor do they provide opportunity to manage competing tasks. Shallice and Burgess (1991) also stated that executive abilities such as planning, organizing, and managing competing demands are the foundation of many everyday life activities, drawing on Norman and Shallice’s (1986) Supervisory Attentional System (SAS) model for their theoretical understanding.

Executive processes are mediated through the frontal lobes, particularly the pre-frontal cortex, and are supported by brain networks involving frontal and parietal gray and white matter structures, as well as subcortical structures including the cerebellum (Bettcher et al., 2016). They are control functions that include self regulation, behavior sequencing and organization, response inhibition, set shifting, working memory, planning and problem solving (Barkley, 2012). Thus, executive functions enable independent, organized, goal-driven behaviors (Stuss, 2011; Lezak et al., 2012) and support performance in non-routine situations that require planning, prioritizing and problem solving (Banich, 2009). Difficulties with executive functioning have been observed in many populations including (but not limited to) traumatic brain injury, stroke, chronic pain (Berryman et al., 2014), non-amnestic MCI (Rosenberg et al., 2011), post-chemotherapy for breast cancer (Yao et al., 2017) and in community dwelling older adults (Royall et al., 2004). Shallice and Burgess (1991) drew on Norman and Shallice’s (1986) SAS model for their theoretical understanding of the MET. In this model, the SAS is the system that monitors plans and actions in non-routine situations and elucidates how routine situations are governed by a contention scheduling system that is fast and automatic (Norman and Shallice, 1986). As the SAS provides for conscious planning and monitoring in non-routine situations, it is slower and more flexible than automatic scheduling and allows for problem solving. The MET was designed to require problem-solving behaviors, plan formulation and monitoring. The MET provides a complex, non-routine situation in that it requires participants to complete a series of real-life tasks (e.g., purchasing items, collecting and writing down information, arriving on time at pre-specified locations) while following a set of predetermined rules (e.g., spend as little money as possible) (Shallice and Burgess, 1991). Thus, the MET was developed based on the SAS as a performance-based, multiple sub-goal scheduling test that taps onto executive abilities such as planning, organizing, and managing competing demands. In its original design, it was administered within a small shopping area: significant performance differences were noted between control participants (*n* = 9) and three adults who had sustained traumatic brain injuries resulting in substantial damage to the frontal lobes (Shallice and Burgess, 1991).

Since its development, multiple versions of the MET have been published including hospital versions (e.g., Knight et al., 2002; Dawson et al., 2009; Morrison et al., 2013), shopping mall versions (e.g., Alderman et al., 2003), virtual reality versions (e.g., Rand et al., 2009; Raspelli et al., 2011; Cipresso et al., 2014; Kizony et al., 2017) and most recently a home version (Burns et al., 2019). The MET-Home is the first version to be published that can be used across different sites without adaptation, greatly enhancing its utility for clinicians and researchers. Other MET versions, such as the shopping center and hospital versions, require the development of site-specific versions to account for the variability in the physical layout, services and retail options in different settings. Site specific adaptations are challenging to develop due to the time required and difficulty ensuring adapted tasks are equitably complex to the tasks in the validated MET versions. Furthermore, the variety of available versions of the MET makes it difficult to compare test results and challenging to rigorously establish properties of validity and reliability. While virtual versions address these issues by providing standardization and uniformity, performance in a virtual world is not the same as performance in the real world (e.g., Claessen et al., 2016; Kimura et al., 2017) although the most recent versions of head-mounted displays are proving more successful, at least for navigation tasks (e.g., Marín-Morales et al., 2019). Nevertheless, in both clinical and research settings, real-world task performance can be informative and there is a need for standardized real-world versions of the MET that could be utilized without site specific adaptations (Nalder et al., 2017).

The development of the MET and other naturalistic tests (see Poncet et al., 2017), has occurred in the context of increasing interest in ecological validity, defined by Sbordone (1996) as “the functional and predictive relationship between the patient’s performance on a set of neuropsychological tests and the patient’s behavior in a variety of real world settings” (p. 16). A full discussion of the pros and cons of laboratory versus naturalistic tasks is beyond the scope of this paper but several points are notable. Laboratory tasks allow for more accurate measurement of specific cognitive processes, as the variance in task performance can be more presicely attributed to the underlying cognitive process of interest, rather than to random error or to other cognitive processes (Snyder et al., 2015). In contrast, naturalistic tasks are conceptually less precise in measuring a specific cognitive domain but their ecological validity is higher (McCoy, 2019). In our view, the MET should not be considered an assessment of executive function *per se* but rather an assessment that allows the effects of executive dysfunction on behavior to be observed and quantified. It was developed in part because Shallice and Burgess (1991) questioned the assumption that cognitive processes measured in experimental settings behave in similar manner when used in a real-world context.

A series of studies have established that the MET is valid, and shown that control participants perform significantly better than individuals with executive dysfunction arising from a variety of etiologies, including acquired brain injury (Knight et al., 2002; Alderman et al., 2003; Dawson et al., 2009; Morrison et al., 2013), polysubstance use (Valls-Serrano et al., 2016), multiple sclerosis (Roca et al., 2008), and schizophrenia and bipolar disorder (Caletti et al., 2013). Administered in a real-world setting (e.g., shopping mall, hospital complex), the MET provides the novelty and unpredictability necessary for characterizing the effect of executive dysfunction on everyday life. In addition, the structure of the MET provides the opportunity to observe individuals’ abilities to plan, problem solve, initiate behaviors, and select and utilize strategies (e.g., ask for help) to assist them in completing the required tasks. Clinicians working with clients with acquired brain injuries have reported that use of the MET provides them with important insights into their clients’ abilities to plan, monitor, control, and adapt to novel situations and to identify challenges they may have with real-world tasks (Nalder et al., 2017). They have also noted that the MET can be a valuable tool in guiding their clinical decision-making related to people’s independence in their communities (Nalder et al., 2017).

To address this need, the Big-Store MET was developed by one of the authors (DJ). This original version of the Big-Store MET includes a set of tasks and rules that could be utilized in one brand of department store commonly found in multiple countries around the world. The aim of the current study was (1) to establish the content validity of the Big-Store MET for use in multiple department stores; (2) to assess the feasibility of testing in a store environment; (3) to determine the inter-rater reliability and internal consistency; and (4) to provide a preliminary characterization of the performance of healthy control participants.

## Materials and Methods

### Research Design

This study had two phases. In phase 1, scientists and clinicians with expertise on the MET were invited to provide feedback on the preliminary version of the Big-Store MET to establish the content validity. In phase 2, the Big-Store MET was trialed with community dwelling adults with no reported history of neurological disease or mental illness (*n* = 14) to assess feasibility (completion within 30 min and practicality of administering within a store environment), determine inter-rater reliability and characterize performance. The study received approval from both Baycrest and University of Toronto Research Ethics Boards and all participants provided informed, written consent.

### Phase 1: Establishing the Content Validity of the Big-Store MET

The original version of the task sheet and rule-list for the Big-Store MET was developed for clinical purposes by one author (DJ) with similar requirements to existing versions such as purchasing items, meeting the examiner at a certain location and time, and writing down specific information (Shallice and Burgess, 1991). The first step in establishing content validity was to determine if it could be used in a variety of department stores (defined as a store that provides a wide-range of consumer goods and services). Two authors (KA, JC) compiled a list of major department store chains operating in multiple geographical locations (across multiple provinces, states and/or countries) and that were located in Toronto where the study was conducted. They visited at least one store from each of the chains to determine the resources and services available within these settings that could inform revisions to the Big-Store MET. More specifically, stores were assessed in relation to the types of items that could be purchased, the number of sub-departments within the store, the number of floors, availability of elevators and escalators, and the presence of a postal counter (as many versions of the MET involve a mailing task).

Based on the resources and services available at these stores, the authors (KA, JC, DD, EN, SR) discussed each item and rule on the preliminary Big-Store MET in relation to whether it was feasible across many sites (see Supplementary Appendix A for task and rule list). In addition, they reviewed the strategies and categorized these according to their previous work in which internal strategies were classified as those that were self-generated and relied on conscious mental manipulations and external strategies as those that relied on cues from the external environment (Bottari et al., 2014). Through this process, a revised version of the original Big-Store MET was developed. Unlike some other versions of the MET that have included site maps (e.g., Knight et al., 2002; Dawson et al., 2009), the Big-Store MET does not include a map as the authors (DD, EN) noted that large department stores seldom have maps for their customers. This revised version and proposed scoring sheet were sent via email to MET experts (*n* = 7) identified as individuals who had previously been involved with the publication of research related to the MET or clinicians who had substantial experience utilizing the MET in their clinical practice.

Identified experts were sent a list of five questions asking them to comment on whether the proposed version of the Big-Store MET was similar to other versions of the MET they were familiar with, whether the cognitive demands for the suggested tasks on the Big-Store MET were are similar to other MET versions, whether they would suggest any changes in the instructions, items and/or rules, and whether they foresaw any challenges with administering the proposed Big-Store MET. All responses were collected and discussed with the research team following which the Big-Store MET was finalized.

### Phase 2: Assessing Feasibility, Determining Inter-Rater Reliability, and Characterizing Performance

#### Participants

For the second phase of the study, participants (*n* = 14) were recruited via word-of-mouth, recruitment flyers posted at community locations, and from the research volunteer database at Baycrest Health Sciences. The inclusion criteria were: age 18–85, able to communicate in English, self-identify as neurologically health (no history of a diagnosed mental illness, acquired brain injury, stroke, or any other neurological or medical condition that could impair components of cognition such as memory, attention, and executive functioning), and able to ambulate without a mobility aid. The age range was wide as executive dysfunction occurs in multiple populations of various ages. Participants were informed at recruitment that they would receive a $10 gift card for study participation.

#### Measures

Demographic data for each participant were collected including age, sex, highest level of educational attainment, ethnicity, handedness, self-perception of overall general health, and self-identified medical conditions. The Montreal Cognitive Assessment (MoCA), a 10-min cognitive screening test, was administered to all participants to provide a characterization of their cognitive status (Nasreddine et al., 2005). The MoCA was administered using the App version^1^. The MoCA has excellent psychometric properties, including high test-retest reliability (*r* = 0.92) and excellent concurrent validity with the Mini Mental State Exam (*r* = 0.87) (Nasreddine et al., 2005). Scores range from 0 to 30 with scores of 26 and above considered normal (Nasreddine et al., 2005).

The Big-Store MET was designed to be conducted in large department stores common to many countries world-wide. Administration of the Big-Store MET followed the same procedure as described in the literature for other versions of the MET. Briefly, raters ensured that the department store had all the required items before participants started the assessment and filled in the task sheet as necessary (e.g., filling in meeting location and time; see Supplementary Appendix A for Task Sheet, Rule List, and Scoring Sheet). At the beginning of the test, examiners reviewed the task and rule list with participants to ensure they understood the task requirements. Participants were provided with the task and rule list on a clipboard with a pen attached which they carried with them, a cell phone (if they did not have their own) and a $10 bill. Questions about the test were answered prior to participants being asked to begin. Once participants had begun to perform the MET tasks, the examiners were careful not to provide additional cues regarding task completion. If the participant directly addressed a question to the examiners, they responded with one of two standard phrases, “*I’ll leave that up to you*” or “*Do your best.*”

During the Big-Store MET, raters followed the participants closely and comprehensively recorded participants’ behaviors including attempt to perform tasks, tasks completioned, tasks omitted, tasks completed partially, rule breaks and strategies used. A debrief interview was conducted with each participant after the completion of the Big-Store MET during which participants were asked about any errors made in relation to task performance or rule breaks. Participants’ responses were recorded.

The test scoring was done after the test was complete. We used the most common methods of scoring described in the literature (Knight et al., 2002; Alderman et al., 2003; Dawson et al., 2009), that include scoring (1) partial task failures including inefficiences (e.g., taking too long to select an item for purchase) and interpretation failures (e.g., where the requirements of the task are misunderstood); (2) rule breaks – where one of the nine explicit rules is broken; and (3) task omissions – where a task is not attempted. Each of these types of errors was given a score of one so that higher scores represent worse performance. Supplementary Appendix A shows the details of the scoring. Additional observations including other performance inefficiencies, social rule breaks and strategies can be noted as clinical observations but due to the wide variety of possible behaviors, we have elected for a more parsimonious scoring method. We and others have noted the difficulty in scoring these behaviors in a reliable way (e.g., Knight et al., 2002; Dawson et al., 2009).

#### Procedure

A large department store in central Toronto (accessible by public transit) that has locations world-wide was selected as the study site. Following completion of phase 1, the finalized Big-Store MET was piloted with two healthy participants to practice the administration and scoring procedures with two trained raters. Two authors (KA, JC) served as the raters. They were trained with administration and scoring using a hospital-based version of the MET prior to scoring the Big-Store MET. Following telephone screening and determination of eligibility, participants met the raters at the department store. Testing was completed in one session of approximately 1 h for each participant including obtaining written consent, administration of the MoCA and Big-Store MET and a final debriefing session.

#### Planned Analysis

Descriptive analyses were conducted for all measures including participant demographics, participant performance, partial task failures, inefficiencies, rule breaks, social rule breaks, and strategies demonstrated by the participants during the Big-Store MET. To determine inter-rater reliability, intraclass correlation coefficients (ICCs) were calculated using the participants’ variance divided by the sum of the participant, rate and error variance. Ninety-five percent confidence intervals were also calculated for each ICC. Calculated ICCs used a two-way random effects, absolute agreement model with two raters (Koo and Li, 2016). Internal consistency was assessed for the total error score using Cronbach’s alpha. All data were analyzed using Version 24.0 of SPSS.

## Results

### Phase 1: Establishing the Content Validity of the Big-Store MET

Four of the seven experts responded to our request for comments on the revised Big-Store MET. All agreed that 8 of the 12 tasks and nine of the nine rules were relevant and appropriate. A summary of the feedback received and revisions made follows. First, in recognition that many department stores do not contain postal counters and/or mailboxes, a suggestion was made to change the mailing task used in many versions of the MET (mailing something to a specified address) to a task requiring the examinee to *return something to customer service*. In addition, experts noted that mailing items is much less commonly done than when the MET was originally devised in 1991. For similar reasons, the task of buying a stamp was changed to buying a toothbrush. *Returning something to customer service* was evaluated through a task analysis as being similar in complexity (number of steps and cognitive demands) to the mailing task. Second, experts suggested using a newspaper as the source of a headline rather than a magazine. However, as magazines are more commonly available in department stores, the task of obtaining a headline from a specified magazine was retained. Third, experts suggested including an interruption task as was first reported by Clark et al. (2017). An interruption task is posited to allow for observation of how participants respond to being taken off task, something that commonly happens in everyday life. The interruption task was revised from the work of Clark et al. (2017) to state, “Please pick up a store flyer and give it to the tester when you complete the exercise. If no flyers are available, tell this to the examiner at the end of the exercise.”

This revised version of the Big-Store MET was then piloted with two participants with no self-reported history of neurological conditions. Ten minutes after the beginning of the test was identified as appropriate for the meeting task as pilot participants took longer than 10 min to complete the Big-Store MET. The final revised version of the Big-Store MET (see Supplementary Appendix A) was used in Phase 2 of the study.

Criteria for stores in which the Big-Store MET can be administered are thus: (1) sell cards and ideally have a value card rack as well as regular card racks; (2) sell magazines (newspapers could be an alternative); (3) have at least two specialty services within the store with specified hours of operation (e.g., vision center, photo center, pharmacy, garden center, etc.); (4) have a return policy that allows for immediate returns after purchase; (5) have baskets and/or trolleys available for customer use. The store used in this study had more than 12 departments (e.g., shoes, groceries, housewares, electronics, etc.) spread over two floors; however, having more than one floor is not a requirement for the Big-Store MET.

### Phase 2: Assessing Feasibility, Determining Inter-Rater Reliability and Internal Consistency, and Characterizing Performance

Fourteen participants were recruited for this study. Their demographic information is shown in Table 1. Within the *other* ethnicity category, one participant identified as Filipino, one identified as Vietnamese, and one as Chinese. Medical conditions disclosed by three of the participants were asthma, Crohn’s disease and idiopathic anaphylaxis. One participant scored below normal on the MoCA (25/30). This person was not excluded as s/he met the inclusion criteria, that is, they did not identify any current or past neurological or mental health condition and the test environment (seated in a mall) may have negatively affected test performance. Seven participants indicated they had previously visited the department store being used as the test location.

**TABLE 1:** Participant Demographics (*n* = 14).

**Variable**	
Age, yr ± SD (range)	27.3 ± 7.9(22−54)
Education, yr ± SD (range)	17.9 ± 1.7(14−21)
Sex	
Female, *n* (%)	10 (71.4)
Male, *n* (%)	4 (28.6)
Ethnicity	
Caucasian, *n* (%)	10 (71.4)
African–American, *n* (%)	1 (7.1)
Other, *n* (%)	3 (21.4)
Handedness	
Right, *n* (%)	13 (92.9)
Left, *n* (%)	1 (7.1)
Self-reported health status	
Good, *n* (%)	12 (85.7)
Excellent, *n* (%)	2 (14.3)
Medical conditions	
No medical conditions, *n* (%)	11 (78.6)
Other medical condition, *n* (%)	3 (21.4)
MoCA Score ± SD (range)	28.9 ± 1.4(25−30)
Number of previous times visiting store ± SD (range)	3.2 ± 6.6(0−25)

*MoCA, Montreal Cognitive Assessment; SD, standard deviation; yr, years.*

Performance on the Big-Store MET is shown in Table 2. Participants completed an average of 10.8 tasks accurately with all participants completing at least nine accurately and no participants omitting tasks. The average total error score was 2.8 with a maximum error score of 6. Time to complete the Big-Store MET varied from 14 to 41 min. One factor appearing to influence time to completion was the length of lines at the cashier and customer service desk.

**TABLE 2:** Performance on Big-Store MET (*n* = 14).

**Score**	**Mean ± SD (range)**
Task completed accurately (out of 12)	10.8 ± 1.2(9−12)
Tasks omitted (out of 12)	0 ± 0
Tasks completed partially	1.2 ± 1.2(0−3)
Specified rule breaks (out of 9)	1.5 ± 1.1(0−3)
Frequency of actual rule break	3.1 ± 12.9(0−10)
**Total errors** (*tasks omitted*+ *partial tasks failures* + *rule breaks*)	2.9 ± 2.2(0−6)
Social rule breaks	0.1 ± 0.3(0−1)
Frequency of social rule breaks	0.1 ± 0.3(0−1)
Time to complete (min)	24.2 ± 7.9(14−41)
Requests for help	2.5 ± 2.1(0−7)
Strategies used	11.5 ± 2.1(10−17)
Frequency of strategies used	30.5 ± 4.5(25−38)

*SD, standard deviation.*

A list of the observed partial task failures, inefficiencies, and rule breaks demonstrated by the participants on the Big-Store MET is shown in Table 3. Of the 52 pre-specified partial task failures, only seven were observed. The most common error was not buying the card from the $1 value card rack, observed in 50% of participants. Participants were observed performing nine other inefficient behaviors including leaving and then returning to a line for no apparent reason and buying an unnecessary item. Of the nine specified rules, four were broken. Nine participants spoke to the examiner, six spent more than the budgeted $8.00 and five went back into side aisles they had previously entered. Only one participant broke a social rule: s/he was observed cutting in front of someone else in the checkout line.

**TABLE 3:** Errors made by neurologically healthy participants on Big-Store MET (*n* = 14).

	**Number of participants who made error**	**Percent**	**Mean frequency of those who made the error ± SD (range)**
**Partial task failures**			*n/a*
∙ Spent excessive time selecting card (>2 min)	2	14.3	
∙ Did not buy card from $1/value rack	7	50.0	
∙ Met examiner at wrong time (<8 min or >12 min)	2	14.3	
∙ Met examiner but did not state the day of the week	1	7.1	
∙ Wrote incorrect closing time	2	14.3	
∙ Gave flyer to examiner before end of exercise	1	7.1	
∙ Did not give the flyer to the examiner	3	21.4	
**Inefficiencies**			
∙ Wandered around an area	1	7.1	*Inefficiencies occurred only once in frequency per participant*
∙ Left a line up and then returned	6	42.9	
∙ Unexplained hesitations during task (>20 s)	1	7.1	
∙ Entered/attempted to enter an unnecessary area (without any clear reason)	1	7.1	
∙ Visited an area and didn’t do anything there	4	28.6	
∙ Bought unnecessary item(s)	1	7.1	
∙ Took obvious steps to looking for unnecessary information/items	2	14.3	
∙ Paid for items separately	1	7.1	
∙ Dropped item(s)	2	14.3	
**Specified rule breaks – of 9 on task sheet**			
∙ Spend no more than $8.00	6	42.9	1 ± 0 (1–1)
∙ You should not go back into a side aisle you have already been in, excluding main aisles	5	35.7	1.2 ± 0.5 (1–2)
∙ You should not complete more than two tasks at customer service	1	7.1	1 ± 0 (1–1)
∙ Do not speak to the examiner *unless* it is part of the exercise	9	64.3	3.3 ± 2.5 (1–9)
**Rule breaks – social**			
∙ Went in front of someone in line	1	7.1	1 ± 0 (1–1)

*Values are mean ± SD (range) or as otherwise indicated.*

The strategies observed as being used by participants are shown in Table 4. All participants used the following seven external strategies: looking overtly at signage or visual landmarks, comparing prices, checking time, checking the task sheet and the rule sheet while walking and/or when stopped. Two additional external strategies frequently used were asking store employees for directions or assistance, used by 12/14 participants and marking tasks as completed on the task sheet used by 10/14. Two internal strategies were also used by the majority (10/12) of participants: using task-oriented self-talk and going to the meeting place early and waiting.

**TABLE 4:** Strategies demonstrated by neurologically healthy participants on Big-Store MET (*n* = 14).

**Strategy**	**Number of participants using the strategy (%)**	**Mean frequency of strategy use/user ± SD (range)**
**Internal strategies**		
∙ Engaged in multitasking	4 (28.6)	1 ± 0 (1–1)
∙ Planned before starting test	4 (28.6)	1 ± 0 (1–1)
∙ Self-talk (task oriented)	10 (71.4)	1.5 ± 0.7 (1–3)
∙ Self-talk (non-task oriented)	5 (35.7)	1.6 ± 1.3 (1–4)
∙ Made notes (other than those required)	3 (21.4)	1.3 ± 0.6 (1–2)
∙ Compared prices	14 (100)	1.8 ± 0.7 (1–3)
∙ Went to meeting place early and waited	10 (71.4)	1 ± 0 (1–1)
**External strategies**		
∙ Organized material and bag	1 (7.1)	1 ± 0 (1–1)
∙ Marked tasks as completed	10 (71.4)	7.2 ± 2.6 (4–11)
∙ Checked change	1 (7.1)	1 ± 0 (1–1)
∙ Separated money from personal money	4 (28.6)	1 ± 0 (1–1)
∙ Check watch	14 (100)	1.3 ± 0.6 (1–3)
∙ Checked task sheet while walking	14 (100)	2.9 ± 1.3 (1–5)
∙ Checked task sheet while stopped	14 (100)	2.9 ± 1.3 (1–5)
∙ Checked rule sheet while walking	14 (100)	2.9 ± 1.3 (1–5)
∙ Checked rule sheet while stopped	14 (100)	2.9 ± 1.3 (1–5)
∙ Asked staff for directions/help/assistance	12 (85.7)	2.9 ± 1.9 (1–7)
∙ Looked overtly at signage/visual landmarks	14 (100)	6.1 ± 2.3 (3–11)

#### Inter-Rater Reliability

Inter-rater reliability was evaluated for eight Big-Store MET sub-scores (see Table 5). Inter-rater reliability was excellent for all sub-scores except frequency of strategy use (ICCs > 0.9 with 95% confidence intervals from 0.74 to 0.99).

**TABLE 5:** Inter-rater reliability.

	**ICC**	**95% confidence interval**
Tasks completed accurately (out of 12)	0.99^∗^	0.96–0.99
Rules adhered to (out of 9)	0.98^∗^	0.95–0.99
Partial task failures	0.99^∗^	0.97–0.99
Total rule breaks (frequency)	0.99^∗^	0.97–0.99
Total errors (*tasks omitted*+ *partial tasks failures* + *total rule breaks*)	0.99^∗^	0.98–0.99
Requests for help	0.98^∗^	0.93–0.99
Strategies used	0.92^∗^	0.74–0.97
Frequency of strategies used	0.35^∗^	0.25–0.75

*^∗^*P* ≤ 0.001.*

#### Internal Consistency

Internal consistency was assessed for the total error score (Σ tasks omitted + partial task failures + number of rules broken). No tasks were omitted by participants and no participant made errors on tasks 1, 3, 4, 5, 8, 9, 10, or 11 (see Big-Met Score Sheet in Supplementary Appendix A for task numbering). Thus, the internal consistency score was based on errors made on four tasks and the number of rules broken. Cronbach’s alpha was calculated as being 0.64.

#### Debrief Interview

After the completion of the Big-Store MET, participants were asked about reasons for errors and rule breaks (see Table 6). All participants were able to provide a rationale for an observed behavior when asked. Misreading, misinterpreting and forgetting rules and tasks were common reasons provided for observed errors and inefficiencies. One observed behavior not included in the table (as it was not related to a specific task or rule) is that one participant used their personal credit card to pay for the items (rather than the $10 provided). This participant explained that this was done so that s/he could use the self-checkout automated cashier in order to be more efficient and skip the long line up. The participant explained they had prioritized finishing the test in the shortest amount of time possible over other considerations.

**TABLE 6:** Participants’ rationale for errors and inefficiencies.

**Test behavior**	**Rationale**
**Partial task failures**	
∙ Did not buy card from $1 rack	∙ Did not see $1 rack. Some participants indicated this led to spending excessive time looking for a card to try to stay within budget
∙ Providing the wrong information on the phone task	∙ Mixed up the information needed for the phone task and the meeting-up task
∙ Late meeting examiner	∙ Forgot about the time
∙ Purchasing an extra item	∙ Misinterpreted the test instructions as s/he thought they needed to have all tasks on the task list to give to the examiner at the end. Purchased an extra item to return to customer service
∙ Did not give examiner the flyer at the end of the test	∙ Forgot. Felt rushed during the task and did not completely read the task
**Specified rules broken**	
∙ Do not speak to the examiner	∙ Forgot the rule. Wanted task clarification and did not think of the questions at the task start
∙ Completed more than two tasks at one location	∙ Forgot the rule, were more focused on finishing than rules
∙ Re-entered a side aisle	∙ Noticed they were going to be over budget and went to exchange card
**Other inefficiencies**	
∙ Entered store areas without doing anything	∙ Did not know store had two levels and were looking for specific items. Indicated store signage was unclear and had gone to area expecting to find a required item
∙ Left cashier line before paying	∙ Left line to find a cheaper card option to stay within budget

## Discussion

This is the first report in the literature of the Big-Store MET, a standardized, community-based version of the MET that has the potential to be used without modification in many department stores in multiple geographic locations. This study establishes the content validity, feasibility of administration and inter-rater reliability with a small sample of community dwelling adults with no known history of neurological or psychiatric disease and provides preliminary information about their performance on this test.

The original version of the Big-Store MET, developed by a clinician, was closely modeled on the original published version by Shallice and Burgess (1991) and similar to other community versions (e.g., Alderman et al., 2003) had 12 tasks, nine rules and was designed to be carried out in a real-world environment. Content validity was investigated through review by research and clinical experts. Thus, it was reflective of things done in everyday life (e.g., purchasing items, finding information, meeting someone at a designated time and place), while following a set of pre-defined rules, all in a real-world environment. The process of expert review established the content validity as the majority of the revised Big-Store MET closely mirrors other versions. Changes made (e.g., including a *return item* task rather than a *mailing task*) were done so only after an activity analysis to ensure comparable complexity and with the principles in mind of maximizing the representativeness of the test to actions undertaken in everyday life and minimizing the need for clinicians and researchers to make site-specific versions. While only a small sample of experts reviewed the preliminary version, two of the authors (DD, EN) have worked extensively with the MET, including publishing a series of papers on the measure (Dawson et al., 2009; Clark et al., 2017; Nalder et al., 2017; Burns et al., 2018, 2019) and teaching workshops on the MET across North America. Thus, we are confident that this process has established the content validity for the revised version of the Big-Store MET.

The two tasks that differ substantially from the original MET are the *interruption task* and the *return task*. We originally introduced the *interruption task* in a revision to the Baycrest MET (Clark et al., 2017) to replace a task reported as ambiguous by many participants in a previous study (Dawson et al., 2009). During this task, participants are interrupted at a set-time after starting the MET and asked to complete an additional task. Thus, they are distracted and have to adjust their plans in relation to completing the overall test to incorporate one more task (the interruption task). We hypothesize that performance on this task may provide insight into participants’ cognitive flexibility, considered a core executive function (Diamond, 2013). This hypothesis is partially substantiated in previous work that showed large correlations between MET scores and inhibition and working memory, both considered components of cognitive flexibility (Dajani and Uddin, 2015). Knight et al. (2002) found a large correlation (*r* = 0.69) between task failures on a hospital MET and the inhibition factor on the Dysexecutive Questionnaire. Similarly, Hansen et al. (2018) reported a large correlation between the task omission score on another hospital MET and that digit span backward. a measure of working memory (*r* = −0.65). In the current study’s sample, all participants completed the interruption task, and were able to carry on with the rest of the assessment without difficulty. This suggests, as expected with control participants, that they may not have had cognitive flexibility difficulties. However, laboratory testing might determine more precisely whether cognitive flexibility was unimpaired.

Our analysis of the *return task* (return an item to customer service) suggested that the complexity of this task was similar to that of mailing something in relation to the planning and number of steps involved and reliance on semantic memory. This task, like the mailing task, involves some financial management and social interaction, both activities highly valued by clinicians using the MET (Nalder et al., 2017). One difference with the *return task* may be that the discussion with a store employee regarding a return is more complex than a discussion to buy a stamp. We hypothesize that this task also requires cognitive flexibility related to shifting attention from one aspect of the task to another.

The Big-Store MET was easily administered. As expected, all participants in this sample completed the test and no participants encountered difficulties with the store employees at any point including when they tried to return an item.

To our knowledge, internal consistency has not been calculated for previous shopping mall versions of the MET. The internal consistency found for the Big-Store MET was somewhat lower than those previously reported (Knight et al., 2002, α = 0.77; Bulzacka et al., 2016, α = 0.94). We suggest that having a sample of only control participants may have resulted in lower internal consistency as there was less variability in performance: the majority of tasks were completed accurately. We noted that relative to our previous studies, many participants in the current study broke the rule of speaking to the examiner. In discussion among the authors, we hypothesized this might have been related to the fact that, due to the recruitment methods, several of the participants knew the testers. Thus, we undertook a *post hoc* analysis of internal consistency omitting this rule from the analysis and found that the alpha increased to 0.71, an acceptable level (Loewenthal, 1996). It will be important to re-assess internal consistency in other samples.

We found the inter-rater reliability on the Big-Store MET to be very high with the exception of that calculated for *frequency of strategies used*. This is in-line with other studies on inter-rater reliability of other versions of the MET that have reported ICCs ranging from 0.71 to 1.00 (Knight et al., 2002; Dawson et al., 2009; Morrison et al., 2013). The finding that inter-rater reliability for the *frequency of strategies used* was very low is not surprising as it is extremely difficult to accurately observe all strategies used. No other studies on the MET have published reliability data on this score. We suggest that frequency of strategy use should be used only to provide qualitative input regarding someone’s performance on the task.

Examination of the performance of this sample of participants on the Big-Store MET may be informative for clinicians and researchers considering using the test. Similar to Alderman et al. (2003) and Dawson et al. (2009), our sample did not show floor effects but ceiling effects (best possible performance, score of 0) may have been present as 5 of 14 participants in our sample had no errors. This performance is somewhat better than that of other control samples (Knight et al., 2002; Alderman et al., 2003; Dawson et al., 2009) possibly because the sample in the current study were volunteers rather than being matched to a clinical sample. Further, scoring variations across studies may also be source of variability. Future research will need to include known groups validity with controls matched to participants from clinical samples, to get a more fulsome picture of control performance on this test.

A final note about the performance of the sample in this study is in relation to completion time which varied widely. We wondered whether this variability related to error scores and/or store familiarity and undertook a *post hoc* analyses of these relationships. However, associations were not significant. Our observations during testing suggest that the variability in time to completion was largely dependent on the day of the week and time of testing. Weekends and evenings appeared to have more shoppers in the department store and resulted in longer wait lines for both the checkout and customer service. Participants with the longest completion time appearted to be those whose testing occurred during these busy time periods.

### Limitations

This study introduces the Big-Store MET and provides preliminary data on performance on this assessment in a small sample of controls who self-identified as neurologically healthy. It is possible, that one or more of our control participants was not aware of, or did not disclose health information that may have influenced their performance. A more comprehensive screening process and baseline testing in future research will help ensure that people with pathology are not included among control participants. This limitation and the small sample size mean that inferences about how other healthy control samples will perform must be made cautiously. As inter-rater reliability was calculated on this sample, it is possible that it will be somewhat lower with a more diverse sample that includes people with neurological pathology. The naturalistic nature of the Big-Store MET means that it provides the “quantifiable analog of the open-ended multiple subgoal situations where … frontal patients would theoretically have problems” (Shallice and Burgess, 1991, p. 728). However, it also means that the cognitive demands of the task vary depending on the business of the store and other unpredictable events.

## CONCLUSION

This study is the first to report a standard, community-based version of the MET, the Big-Store MET. The study establishes preliminary content validity and inter-rater reliability and shows that the test is feasible to administer. The development of the Big-Store MET provides a foundation for future psychometric work and introduces a version of the MET to the clinical and research community that we believe will be an asset in identifying everyday life difficulties related to executive dysfunction.

## Data Availability Statement

The raw data supporting the conclusions of this manuscript will be made available by the authors, without undue reservation, to any qualified researcher.

## Ethics Statement

The studies involving human participants were reviewed and approved by Baycrest Research Ethics Board, Baycrest Health Sciences, Toronto, and the University of Toronto Research Ethics Board, Toronto. The patients/participants provided their written informed consent to participate in this study.

## Author Contributions

DD and EN conceived the study and methods. DJ conceived the idea of the Big-Store MET and developed a preliminary version. DD and SR oversaw the data acquisition and analyses. KA and JC carried out the study, conducted the analyses, and wrote the first draft of the manuscript. All authors approved the final version for submission and are accountable for all aspects of the work.

## Conflict of Interest

The authors declare that the research was conducted in the absence of any commercial or financial relationships that could be construed as a potential conflict of interest.

## Footnotes

1 https://www.mocatest.org/
