# Supplementary material for: Developing and Validating a Big-Store Multiple Errands Test
Source: Front Psychol. 2019 Nov 15;10:2575. doi: 10.3389/fpsyg.2019.02575 (PMC6873776; doi:10.3389/fpsyg.2019.02575)
Supplement: Supplementary file 1 [file Data_Sheet_1.pdf]

Supplementary Appendix A: Big-Store Multiple Errands Test

In this exercise you should complete the following tasks:

You should do the following 5 things:
- Buy a bottle of Coca-Cola
- Buy a birthday card
- Phone <*insert name*> at <*insert phone #*> and say who you are, where you are & the weather today
- Buy a toothbrush
- Return (one of the purchased items[1]) at customer service

You must meet me at (or in front of) the <*insert location, e.g., Pharmacy*>at <*insert specific time, 10 minutes from start to task*>and tell me what day of the week it is.

You should obtain the following information and write it down in the spaces below:
- What is the closing time of the <*insert name of department within store, e.g., Pharmacy*> department on a Wednesday ____________?
- What is the opening time of the <*insert name of department within store, e.g., Vision Centre*> department on a Tuesday ___________?
- What is the price of a large bag of chips ____________?
- What is the (biggest) headline of <*insert name of popular magazine or newspaper? readily available at store*> ____________?

Tell the person observing you when you have completed the exercise.

While carrying out this exercise you must obey the following rules:
- You should carry out all these tasks but may do so in any order
- You should spend as little as possible and no more than $_________.
- You should stay within the store and parking lot adjacent to this store
- You should not enter any "staff only" areas
- You should not go back into a side aisle you have already been in, excluding main aisles
- You should not complete more than 2 of the tasks at customer service
- Do not enter a department unless it is related to a task
- Take as little time to complete this exercise as possible without rushing excessively
- Do not speak to me unless this is part of the exercise

Interruption task (Written on separate sheet of paper, given to the participant 5 minutes into the test, saying, "Excuse me there is one more thing I need you to do."):

Please pick up a store flyer and give it to the examiner when you complete the exercise. If no flyers are available, tell this to the examiner at the end of the exercise.

---

[1] Suggest adding this phrase for purposes of clarification.

Big-Store MET Score Sheets

Task Order	Tasks	Task Completed (Y/N/P)	Task Errors	Observed
	1. Buy a bottle of Coca-Cola		Bought drink other than Coca-Cola	
			Spent excessive time looking for Coca-Cola (>2min)	
			Exchanged drink while in line or at cash (reason?)	
			Buy a canned drink rather than a bottle.	
	2. Buy a birthday card		Bought wrong type of card	
			Spent excessive time selecting card (>2min)	
			Did not buy from $1/value rack	
			Exchanged card while in line (reason?)	
	3. Dial correct phone # and give specified information		Needed to hang up and redial	
			Called wrong person	
			Gave incorrect information on phone call	
			Gave too much/ too little information	
	4. Buy a single toothbrush		Bought item other than a toothbrush	
			Bought the wrong number of toothbrushes	
	5. Return one item at customer service		Unable to identify/decide which item to return or spent excessive time returning item (>2min)	
			Returned more than one item	
			Opened/used item before returning it	
			Forgot to provide receipt/lost receipt	
			Forgot to collect change/check change/put change in safe location	
	6. Meet at specified location		Met examiner at the wrong place	
			Met examiner at the wrong time (< 8 min or > 12 min)	
			Met examiner but did not mention current day of the week	
			Wrote the day instead of telling the examiner the day	
			Met the examiner and stated wrong info (e.g., weather, time) instead of day	
	7. Closing time of the Vision Centre on a Wednesday		Wrote incorrect closing time	
			Wrote closing time for the wrong place	
			Wrote opening time or opening & closing time	

			Wrote correct time only by chance (e.g., did not actually check the hours)	
			Orally stated closing time instead of writing it down	
	8. Opening time of the Photo Centre on a Tuesday		Wrote incorrect opening time	
			Wrote opening time for the wrong place	
			Wrote closing time or closing & opening time	
			Wrote correct opening time by chance (e.g., did not actually check the hours)	
			Orally stated opening time instead of writing it down	
	9. Price of large bag of chips		Wrote the incorrect price	
			Wrote the price for another type of product	
			Wrote the correct price only by chance	
			Spent excessive time find the item (>2 min)	
			Bought item	
			Orally stated price instead of writing it down	
	10. Headline of today's (People) Magazine		Wrote the headline of another magazine	
			Wrote other information that isn't the headline	
			Orally stated headline instead of writing it down	
	11. Tell the examiner when finished		Thought meeting and task end were one/same	
			Did not explicitly tell examiner when they were finished	
	12. Interruption task Pick up flyer and hand it to examiner after the test.		Picked up the wrong item	
			Gave flyer at the incorrect time	
			Showed flyer but did not give to examiner	
			Did not show or give flyer to the examiner, or did not state that none were available	

Legend: Y=task completed with no errors; N=no part of task attempted=task omitted; P=task attempted but task error observed.

**Rules**	**Adhered To**	**Broken (note frequency)**
1. All tasks carried out and, in any order		
2. Spend no more than $8.00		
3. Stay within the limits of the store area and parking lot adjacent to the store		
4. Do not enter any "staff only" areas		
5. You should not go back into a side aisle you have already been in, excluding main aisles		
6. You should not complete more than 2 of the tasks at customer service		
7. Do not enter a department unless it is related to a task		
8. Take as little time to complete this exercise as possible without rushing excessively		
9. Do not speak to the examiner unless it is part of the exercise		

Total Error Score= #tasks omitted + number of partial task failures + number of rules broken

OBSERVED INEFFICIENCIES, SOCIAL RULE BREAKS & STRATEGY USE

**Inefficiencies**	**Observed (Y/N)**	**Frequency**
Wandered around an area		
Left a line up and then returned		
Unexplained hesitations during task (>20 sec)		
Unnecessary conversations with individuals		
Entered/attempted to enter an unnecessary area (without any clear reason)		
Visited an area and didn't do anything there		
Unnecessarily sat down		
Bought unnecessary item(s)		
Repeated question(s)		
Took obvious steps to look for unnecessary information/items		
Paid for items separately		
Paid for items with personal money or credit card		
General awkwardness/disorganization with task items		
Dropped item(s)		
Asked non-staff for directions/help/assistance		
Verbally indicated wanting to 'give up'/'quit'		
**Observed Social Rule Breaks**		
1)		
2)		
**Strategies**	**Observed**	**Frequency**
**Internal Strategies**<br>• Engaged in multitasking		
• Planned before starting test		
• Self-talk (task oriented)		
• Self-talk (non-task oriented)		
• Compared prices		
• Went to meeting place early and waited		
**External Strategies Created**<br>• Made notes (other than those required)		
• Organized material and bag		
• Marked tasks as completed		
• Checked change		
• Separated money from personal money		
• Check watch		

• Checked Task sheet while walking		
• Checked Task sheet while stopped		
• Checked Rule sheet while walking		
• Checked Rule sheet while stopped		
• Asked staff for directions/help/assistance		
• Looked overtly at signage/visual landmarks		
